# Factors Predicting Practices in Prevention of COVID-19 and Impacts among Population in Chiang Mai, Thailand

**DOI:** 10.3390/medicina58040505

**Published:** 2022-04-01

**Authors:** Nongyao Kasatpibal, Peninnah Oberdorfer, Wasan Katip, Raktham Mektrirat, Usanee Wattananandkul, Kwaunpanomporn Thummathai

**Affiliations:** 1 Division of Nursing Science, Faculty of Nursing, Chiang Mai University, Chiang Mai 50200, Thailand; kwaunpanomporn.th@cmu.ac.th; 2 Epidemiology Research Group of Infectious Disease (ERGID), Chiang Mai University, Chiang Mai 50200, Thailand; aoberdor@med.cmu.ac.th (P.O.); wasan.katip@cmu.ac.th (W.K.); raktham.m@cmu.ac.th (R.M.); usanee.anukool@cmu.ac.th (U.W.); 3 Department of Pediatrics, Division of Infectious Diseases, Faculty of Medicine, Chiang Mai University, Chiang Mai 50200, Thailand; 4 Department of Pharmaceutical Care, Faculty of Pharmacy, Chiang Mai University, Chiang Mai 50200, Thailand; 5 Department of Veterinary Bioscience and Veterinary Public Health, Faculty of Veterinary Medicine, Chiang Mai University, Chiang Mai 50200, Thailand; 6 Department of Medical Technology, Division of Clinical Microbiology, Faculty of Associated Medical Sciences, Chiang Mai University, Chiang Mai 50200, Thailand

**Keywords:** predictor, practice, prevention, impact, COVID-19

## Abstract

*Background and objectives:* The pandemic of COVID-19 is a global concern requiring urgent and effective action. However, the data on prevention practices and the impact of COVID-19 among the Thai population have not been clearly described. This study aimed to examine the knowledge, attitudes, perception, practices, and factors predicting practices in the prevention of COVID-19 and to study the impact of COVID-19 on people’s livelihoods. *Materials and Methods*: A cross-sectional study was performed between April and November 2020. A questionnaire eliciting demographic data and information on knowledge, attitudes, perception, prevention practices, and impact of COVID-19 was given to 500 people who lived in Chiang Mai, and 480 usable questionnaires were returned, for a response rate of 96.0%. Data were analyzed using descriptive statistics and multivariate linear regression. *Results*: Less than half of the participants had a high level of knowledge (45.4%) about COVID-19. Most of them had a high level of attitudes (95.6%), perception (72.1%), and prevention practices (90.4%). Female (β = 0.11, *p* = 0.006), patient status (β = 0.17, *p* < 0.001), knowledge (β = −0.10, *p* = 0.020), attitudes (β = 0.37, *p* < 0.001), and perception (β = 0.21, *p* < 0.001) about COVID-19 prevention were the predicting factors for overall prevention practices (R^2^ = 0.288). Most participants perceived the overall impact of COVID-19 at moderate and high levels (47.1 and 37.8%, respectively). The highest impact was an economic burden, followed by psychological, social, and physical impacts. *Conclusions*: Policymakers should enhance attitudes and perception about COVID-19 prevention to improve the COVID-19 prevention practices. This may help to reduce the new cases of COVID-19 and may result in reducing the impact of COVID-19 on people’s livelihoods.

## 1. Introduction

COVID-19 is an emerging disease that was first discovered in late 2019 in Wuhan, Hubei Province, China [1]. COVID-19 is rapidly spreading worldwide because most infected people have no signs and symptoms [2]. The reproduction number (R0) of COVID-19 was found to vary between 2.4 and 8.0 [3]. Data on 17 March 2022, found that more than 646 million people have been infected with COVID-19 worldwide, with about 6 million deaths [4]. In Thailand, there have been 3.2 million infected patients and 23,998 deaths [5]. In northern Thailand, Chiang Mai province had the highest number of cases and deaths, with 51,316 infected patients and 213 deaths [6]. Chiang Mai is the largest city in the northern region and the most popular tourist destination in northern Thailand. There were 8.7, 9.3, and 9.6 million tourists in 2014, 2015, and 2017, respectively [7], and 33% are foreign tourists [8]. During the first COVID-19 outbreak in the year 2020 through March 2022, the tourism business in Chiang Mai faced an enormous decrease in number of tourists. However, Chiang Mai, Phuket, and Bangkok were reported as the top three domestic destinations in the period following the COVID-19 outbreak [9]. Chiang Mai University (CMU) is also the largest university in the northern part of Thailand and one of the leading universities in the country. The university employs 2200 teachers and professors and educates more than 30,000 students [10]. Though these university students come from different places, most of them (undergraduates) spend at least four years in Chiang Mai. If local and inhabitant people perform incorrect practices in preventing COVID-19 infection, it could lead to a new wave of COVID-19 epidemic and pandemic. Therefore, it is intriguing to investigate whether the people living in Chiang Mai, including health science students, have the knowledge and awareness of the correctness of practices in preventing infection and spread of COVID-19.

Previous studies demonstrated that the practices in the prevention of COVID-19 are different according to knowledge [11], attitudes [12], sex, age, education level, occupation [13], and COVID-19 perception [14,15]. According to the cognitive–affective–behavioral theory, increased knowledge relates to attitude and practices. The theory is founded on the idea that expanding one’s knowledge will lead to a change in behavior [16]. In addition, females are more concerned with health care and disease prevention practices than males. As people age, they gain more outcome experience from incorrect practices. Higher education improves knowledge, which influences attitudes and behaviors [17]. The perception of COVID-19 information influenced a positive attitude and proper COVID-19 prevention measures, leading to an increase in COVID-19 prevention behaviors [18]. However, the related factors and correlation coefficient or predicting factors were specific in each study and country.

Few studies examined the factors predicting practices in the prevention of COVID-19, especially in the Thai context. The results obtained provided inadequate information for the policymakers to implement policies to promote and monitor the correct practices in preventing COVID-19.

The impact of the COVID-19 pandemic is another important issue because it affects the economic, social, educational, religious, cultural, and psychological aspects of the COVID-19 epidemic. It is contributing to the economic slowdown of countries around the world [19] and reduced gross domestic product (GDP) and economic growth [20]. Essential everyday products are affected [21]. It is expected that such situations may have serious impacts by causing poverty for up to 420–580 million people in the world [22]. It also affects people’s daily activities. In addition, many infected people are stigmatized. People are afraid, panicked, and anxious that the infected person will spread the infectious disease to themselves, which can affect the occurrence of disharmony in society [23]. Many studies have found that the COVID-19 epidemic affects the education of students at all levels because both students and teachers are not well prepared for online teaching and learning [24]. However, there are few studies on the impact of COVID-19 in Thailand during the study period and the impact in terms of magnitude and effects may be different from other countries, regions, or communities. We, therefore, conducted this study (1) to examine the knowledge, attitudes, perception, and practices in the prevention of COVID-19; (2) to examine the factors predicting practices in the prevention of COVID-19; and (3) to study the impact of COVID-19 on the population in Chiang Mai province.

## 2. Materials and Methods

### 2.1. Study Design and Participants

A cross-sectional study was performed between April and November 2020. The convenience sampling method was used to select the participants. The inclusion criteria were those aged 18 years old and above who lived in Chiang Mai province and had the ability to communicate in the Thai language and were willing to cooperate in this study. The exclusion criteria were people who had a serious illness and inability to provide information for this study.

### 2.2. Ethical Considerations

This study was approved by the Research Ethics Committee at the Faculty of Nursing, Chiang Mai University (reference No. 048-2020), and the Research Ethics Committee at the Faculty of Medicine, Chiang Mai University (reference No. 137-2020). The participants were informed about the study’s objectives, procedures, and benefits before signing the informed consent form. Data were collected after the participant had given his or her permission, and the participant’s identity was kept confidential.

### 2.3. Instrument

Research instruments developed by the researchers included a questionnaire that consisted of six parts: (1) A demographic data questionnaire consisted of gender, age, education level, occupation, travel to risk areas, quarantine experience, and signs and symptoms of COVID-19 infection. (2) A knowledge test measured knowledge related to COVID-19 and preventing COVID-19 infection; it was a four-choice test consisting of 20 questions, and the total score was 20 points. (3) An attitude questionnaire measured attitudes toward COVID-19 prevention, containing a total of 26 items with a 4-point rating scale ranging from strongly disagree (1 point) to strongly agree (4 points). (4) Perception of COVID-19 was measured by a questionnaire with a total of 28 items with a 4-point rating scale ranging from never (1 point) to always (4 points) perceived correct information. (5) A practice questionnaire measured the COVID-19 infection prevention practice, containing a total of 35 items with a 4-point rating scale ranging from never (1 point) to every time (4 points). (6) The impact of COVID-19, including daily life activity, mental, emotional, social, and economic impacts, was assessed with a total of 17 items with a 4-point rating scale ranging from low impact (1 point) to very high impact (4 points). The research instrument was validated by six experts. The content validity index values for the practice questionnaire, the knowledge test, the attitude questionnaire, the perception about COVID-19 questionnaire, and the impact of COVID-19 questionnaire were 0.98, 0.98, 0.99, 0.99, and 1.00, respectively, and reliability values were 0.76, 0.86, 0.92, 0.96, and 0.94, respectively.

### 2.4. Data Collection

A questionnaire eliciting demographic data and information on knowledge, attitudes, perception, prevention practices, and impact of COVID-19 was contributed to 500 people who lived in Chiang Mai, and 480 usable questionnaires were returned, for a response rate of 96.0%. The participants were 320 university students and staff at Chiang Mai University (CMU) and 160 patients who waiting for laboratory tests or physical examinations or the hospital billing and payment process at the outpatient department (OPD) at CMU Hospital. The students and staff were selected from five faculties, namely Medicine, Nursing, Pharmacy, Associated Medical Sciences, and Veterinary Medicine. These medical professionals are expected to be knowledgeable on the practice and prevention of COVID-19-infected persons and were more likely to experience those infected with a new strain of COVID-19. The researcher invited a sample group of students and staff of CMU with posters inviting them to join the research project in front of the elevator of the school building in five participating faculties. For the patients, the researcher posted a poster inviting them to join the research project at the 1st floor hall of the CMU Hospital. If members of the sample group were interested, they could apply for the research project and answer the questionnaire by scanning the QR code provided on the invitation poster.

### 2.5. Data Analysis

R version 3.5.1 was used to analyze the data. Demographics, knowledge, attitudes, perception, prevention practices, and impact of COVID-19 data were calculated as frequency and percentage, mean and standard deviation, and median and range as appropriate. Potential predicting factors for practices in the prevention of COVID-19 included sex, age, education, status, history of traveling to a high-risk country, history of traveling to high-risk areas in Thailand, close contact with high-risk groups, close contact with COVID-19 patients or patients under investigation, having signs or symptoms of COVID-19, quarantine experience for COVID-19, knowledge, attitude, and perception. Each variable was analyzed by using simple linear regression. All significant predicting factors were then included in the multivariate linear regression where wearing a mask and coughing etiquette, practicing hand hygiene, avoiding crowded places, practicing health promotion, and overall prevention practice were treated as separate dependent variables. The backward stepwise multivariate linear regression was performed to identify predictors for overall prevention practices and subgroups of COVID-19 prevention practices. The level of significance was set at *p* < 0.05.

## 3. Results

### 3.1. Demographics

Out of a total of 500 questionnaires, 486 questionnaires were returned. Of these, six were excluded because they were incomplete, leaving 480 usable questionnaires, for a 96.0% response rate. The majority of the participants were female (79.4%), and the mean age was 37.7 + 15.8 years. Most participants were university students (33.5%) and university employees (29.6%). Most of them held a bachelor’s degree (49.3%). Most of the participants did not travel abroad (97.8%) or domestically (85.0%). Most of them were not considered high-risk people for COVID-19 infection. Only 18.1% of participants had experienced quarantine (Table 1).

### 3.2. COVID-19 Knowledge, Attitudes, Perception, and Prevention Practices

Less than half of the participants were highly knowledgeable (45.4%), and 15.4% of them had a low level of knowledge. Most of them had a high level of attitudes towards COVID-19 prevention (95.6%), had a high level of overall perception about COVID-19 information (72.1%), and had a high level of overall COVID-19 prevention practice (90.4%) (Table 2).

More than 90.0% of participants always performed the following prevention practices: wearing a mask by bending the nose wire over the nose to fit close to the face (93.3%), wearing a mask when going to crowded places (92.7%), wearing a mask with the white side facing inward and the colored side facing outwards (92.7%), and wearing a mask by completely cover the nose, mouth, and chin (91.5%). However, nine practice items were always performed by less than 50.0% of the participants. The prevention practice item always practiced by the lowest proportion of participants was cleaning frequently touched surfaces, such as beds, tables, chairs, and objects around the bathroom, with bleach (31.5%) (Table 3).

### 3.3. Factors Predicting Practices in Prevention of COVID-19

Female (β = 0.11, *p* = 0.006), patient status (β = 0.17, *p* < 0.001), knowledge about COVID-19 (β = −0.10, *p* = 0.020), attitudes toward COVID-19 prevention (β = 0.37, *p* < 0.001), and perception about COVID-19 information (β = 0.21, *p* < 0.001) were the predicting factors for overall prevention practices (R^2^ = 0.288). Predicting factors for subgroups of COVID-19 prevention practices, including wearing a mask and coughing etiquette (R^2^ = 0.218), practicing hand hygiene (R^2^ = 0.157), avoiding crowded places (R^2^ = 0.219), and practicing health promotion (R^2^ = 0.256), are also provided (Table 4).

### 3.4. Impact of COVID-19

Most participants perceived the overall impact of COVID-19 at moderate and high levels (47.1 and 37.8%). The highest effect was economic impact, followed by psychological, social, and physical impacts, in that order. More than half of the participants had economic and psychological impacts at high levels (66.7% and 54.0%, respectively) (Table 5).

### 3.5. Response to COVID-19 Knowledge, Attitude, Perception, and Impact Items 

More than 90.0% of participants had correct knowledge about the most common symptoms of COVID-19 (97.5%), preventing the spread of COVID-19 by wearing a mask when going out (97.1%), and surveillance and observing the possible illnesses of COVID-19 for 14 days (96.0%). The item answered correctly by the lowest proportion of participants was the question regarding whether patients under investigation for COVID-19 might spread the coronavirus to other people (41.7%) (Supplement Table S1).

The top three positive attitudes toward COVID-19 prevention among the participants were wearing the mask correctly (84.6%), wearing a mask when going to crowded places (83.5%), and avoiding crowded elevators could reduce the risk of COVID-19 infection. However, only 47.3% of them thought that using tissue paper to cover the mouth and nose when coughing and sneezing reduces the risk of COVID-19 spread (Supplement Table S2).

The participants responded that they always perceived correct information about the preventative measures (79.0%), mode of transmission (72.3%), and the list of high-risk countries/areas (68.3%), but only 45.0% of them always obtained the correct information about the number of patients under investigation (PUIs). The participants perceived that the Thailand Department of Disease Control (DDC) website (66.3%), World Health Organization (WHO) website (62.9%), and television (51.9%) were the top three sources that always provided correct information, whereas the lowest one was neighbors (12.1%) (Supplement Table S3).

About half of the participants perceived that COVID-19 resulted in economic impact at a very high level for the cost of masks (56.7%) and hand sanitizer (53.1%). More than one-third of them were unemployed and out of work temporarily (38.1%). Some participants had a high level of stress (20.6%), discrimination (24.0%), and insomnia (10.8%) (Supplement Table S4).

## 4. Discussion

This study demonstrated that less than half of the participants had a high level of knowledge, and some of them were classified as low level. This may be because COVID-19 is a novel emerging disease, some knowledge of the disease may be unclear, and some participants may not have a deep knowledge of COVID-19. The participants had a low score for the knowledge about how PUIs for COVID-19 might spread coronavirus to other people, and if hands were visibly soiled or contaminated with saliva or respiratory secretions, could not use an alcohol-based hand rub. These may result in malpractice in wearing a mask, performing hand hygiene, and keeping physical distancing, leading to an increase in the spread of COVID-19. However, this study found that knowledge had a negative correlation with practices, but attitudes and correct perception of COVID-19 information were positively associated with practices. The findings from this study are inconsistent with another study which found that knowledge directly influenced both attitudes and practices and efficacy belief mediated the relationship between knowledge and preventive practices of COVID-19 [25]. The results suggested that the enhancing of attitudes and correct perception may help to improve knowledge and practices of COVID-19 prevention.

Several studies [26,27] showed that some sources of information are unreliable and a large amount of misleading and false information or fake news about COVID-19 is shared, especially among social media users [26,27]. Another study reported that the links comprising fake news were shared 2.3 million times (23.1% of the total shares) [28]. Some participants in this study may receive fake news which may lead to developing incorrect knowledge, misperception, and incorrect practices in the prevention of COVID-19. It may also affect the spread or new wave of COVID-19 outbreak. Everyone should be a part of spotting and combatting fake news and misinformation together with providing reliable sources of COVID-19 information. This study demonstrated that the proportion of participants who always perceived correct information about COVID-19 ranged from 45.0% (information about the number of PUIs) to 79.0% (information about the preventative measures). This reflects that 21.0% to 55.0% of contributors may perceive untrue information or fake news. They acknowledged that the Thailand DDC website, WHO website, and television were the top three reliable sources of COVID-19 information and that neighbors might have a high chance to convey unreliable information to the participants. Their neighbors might not intend to provide misinformation to the participants, but they may view fake news on social media and not check before sharing the information [26,27,28].

Almost all participants had a high level of attitudes toward COVID-19 prevention. In addition, attitudes had a positive relationship with overall prevention practices and four subgroups of practicing (wearing the mask and coughing etiquette, practicing hand hygiene, avoiding crowded places, and practicing health promotion). This study confirmed the findings from the previous studies that attitudes significantly influenced COVID-19 prevention practices [25,29,30,31]. This is because attitudes toward the behaviors affected the intention to perform certain behaviors such as wearing the mask and hand hygiene.

Although most participants had a high level of overall prevention practices, only 30.0% to 50.0% of them could perform correct processes of several practice items such as correct steps and duration of performing hand hygiene, the distance for physical distancing, the steps and frequency of environmental cleaning, and the steps of wearing an N95 mask. In addition, practicing health promotion and personal hygiene were performed correctly by about 50% to 60% of participants. These are the gaps in prevention practices and the opportunities for improvement. Promoting correct attitudes, perception, and knowledge through comprehensive training, role models, and creating a learning organization and safety culture may help to improve correct practices in the prevention of COVID-19.

This study also displayed the positive relationship between females, increasing age, student and patient status, and prevention practices. Women are more likely than men to follow guidelines to prevent the spread of COVID-19 such as properly wearing masks, performing hand hygiene, and avoiding contact with sick people [32]. In addition, the attitudes and values increase with years of age and experience. Most students study from home in accordance with the work/study from home policy. This may result in them avoiding crowded places more than university staff and patients because most students in Thailand have responsibility only for studying whereas the others may have several responsibilities such as going to the market to buy food and going to the hospital for a physician appointment. Most patients recognized that they are at high risk of COVID-19 infection. They, therefore, practice hand hygiene and health promotion more than students and university staff to reduce the spread of the virus and promote immunity.

The economic burden is the highest impact on the participants due to the increasing cost of masks and hand sanitizer and decreasing incomes (or lack thereof). This results in psychological problems such as stress. Other profound impacts are discrimination and insomnia because they will increase stigmatization and decrease the quality of life. These findings are consistent with previous studies [15,16,17,18,19]. However, the priority of problems and the strategies to solve them may vary among the countries or regions. Qualitative research should be conducted to explore the root causes and identify appropriate strategies for this population. Policymakers should implement proper and timely interventions to resolve these problems. Moreover, we suggest that the government, interested stakeholders, and the media continue to work on COVID-19′s spread to improve society’s awareness and mindset, resulting in better COVID-19 prevention practices.

One Health is a well-known concept; it has remained on the periphery of most operational health plans rather than being the focus. Even though worldwide experts and policymakers have agreed on this principle, the transfer from vision to practice has been slow. The COVID-19 pandemic has wreaked havoc on the global economy and continued to endanger human lives around the planet. The Achilles heel of our health policy has shown to be a lack of understanding of the concepts of the One Health approach in the current health care system. Short-term preventive methods such as social isolation, lockdown, and hand hygiene are used by countries all over the world, but they are difficult to maintain in the long run. As a result, it is long past time for us to abandon our one-dimensional approach to disease control and prevention. For effective control of the current harsh situation, logical implementation of the One Health approach should be our primary consideration [33].

There are some limitations of our study. First, this study was based on a single province in Thailand, the findings might not be applicable to other settings unless the circumstances are identical. Second, all participants were voluntary, and convenience sampling was used, which may lead to selection bias. Third, all participants had some level of Thai language communication ability. Therefore, the results of this study might not be generalized to people not able to communicate using the Thai language. Fourth, two-thirds of participants were university students and staff. The findings might not be a good representative of laypeople who have lower education than the participants. Finally, data were collected using a self-report questionnaire, which has the potential to introduce information bias. The questionnaire used in this study, on the other hand, had high content validity, and all participants were instructed how important it was to answer the questions exactly.

## 5. Conclusions

Less than half of the participants had a high level of knowledge about COVID-19. Most of them had a high level of attitudes, perception, and prevention practices. Female, patient status, attitudes, and perception about COVID-19 prevention were the preventive predicting factors for overall prevention practices, but knowledge had a negative relationship with the practices. Most participants perceived a moderate impact of COVID-19. The highest impact was an economic burden, followed by psychological, social, and physical impacts. Policymakers should enhance positive attitudes and correct perceptions about COVID-19 prevention to improve COVID-19 prevention practices. Success in promoting correct practices may result in the reduction in new cases and the impact of COVID-19 on people’s livelihoods.

## Figures and Tables

**Table 1:** Demographic characteristics of participants (*n* = 480).

Characteristics	*n*	%
Sex		
Female	381	79.4
Male	99	20.6
Age (years)		
≤20	8	1.7
21–30	208	43.3
31–40	80	16.7
41–50	77	16.0
51–60	49	10.2
>60	58	12.1
Mean = 37.7, SD = 15.8; Median 33.5, Range = 19–88		
Education		
Primary school	23	4.8
Secondary school	139	29.0
Bachelor degree	237	49.3
Master degree	56	11.7
Doctoral degree	25	5.2
Status		
University student	163	34.0
University employee	157	32.7
Patient	160	33.3
History of traveling to high-risk country		
No	469	97.8
Yes	11	2.2
China	3	27.3
Japan	3	27.3
Others *	5	45.4
History of traveling to high-risk area in Thailand		
No	408	85.0
Yes	72	15.0
Bangkok	30	41.7
Other provinces	42	58.3
Close contact with high-risk group		
No	466	97.1
Yes	14	2.9
Close contact with COVID-19 patient or patient under investigation		
No	448	93.3
Yes	32	6.7
The participant had signs or symptoms of COVID-19		
No	394	82.1
Yes	86	17.9
Sneeze	10	11.6
Fever	7	8.1
Cough	4	4.6
Muscle aches	8	9.3
Diarrhea	8	9.3
Runny nose	4	4.6
Dyspnea/shortness of breath	4	4.6
Sore throat	3	3.6
2 signs or symptoms	18	20.9
3 signs or symptoms	12	14.0
4 signs or symptoms	5	5.8
5 signs or symptoms	3	3.6
The participant had been quarantined for COVID-19		
No	393	81.9
Yes	87	18.1

* America, Scotland, Malaysia, and Canada.

**Table 2:** Level of COVID-19 knowledge, attitude, perception, and prevention practices among participants (*n* = 480).

Score	Level	*n*	%
Knowledge about COVID-19			
≥12	Low	74	15.4
13–16	Moderate	188	39.2
17–20	High	218	45.4
Mean = 15.6, SD = 2.9; Median = 16, Range = 5–20			
Attitude toward COVID-19 prevention			
26–51	Low	0	0
52–77	Moderate	21	4.4
78–104	High	459	95.6
Mean = 95.2, SD = 9.0; Median = 98, Range = 60–104			
Overall perception about COVID-19			
0–25	Low	0	0
26–50	Moderate	134	27.9
51–75	High	346	72.1
Mean = 57.3, SD = 9.8; Median = 58.0, Range = 26–75			
Perceived information about COVID-19			
0–13	Low	1	0.2
14–26	Moderate	64	13.3
27–39	High	415	86.5
Mean = 33.3, SD = 5.2; Median = 34.0, Range = 13–39			
Source of information about COVID-19			
0–12	Low	22	4.6
13–24	Moderate	231	48.1
25–36	High	227	47.3
Mean = 23.9, SD = 6.4; Median = 24.0, Range = 5 – 36			
Overall COVID-19 prevention practice			
0–42	Low	1	0.2
43–84	Moderate	45	9.4
85–126	High	434	90.4
Mean = 104.8, SD = 13.9; Median = 106.0, Range = 39–126			
Wearing a mask and coughing etiquette			
0–12	Low	0	0
13–24	Moderate	44	9.2
25–36	High	436	90.8
Mean = 31.0, SD = 4.3; Median = 32.0, Range = 14–36			
Practicing hand hygiene			
0–11	Low	2	0.4
12–22	Moderate	94	19.6
23–33	High	384	80
Mean = 26.6, SD = 5.1; Median = 27.0, Range = 10–33			
Avoiding crowded places			
0–4	0–4	0–4	0–4
5–9	5–9	5–9	5–9
10–12	10–12	10–12	10–12
Mean = 9.6, SD = 2.2; Median = 10.0, Range = 1–12			
Practicing health promotion			
0–15	Low	1	0.2
16–30	Moderate	54	11.3
31–45	High	425	88.5
Mean = 37.6, SD = 5.7; Median = 38.0, Range = 8–45			

**Table 3:** Response to COVID-19 prevention practice items among participants (*n* = 480).

Prevention Practice Items		Level of Prevention Practice			
		Never	Sometimes	Usually	Always
		*n* (%)	*n* (%)	*n* (%)	*n* (%)
1.	You wear a mask by bending the nose wire over your nose to fit close to your face	0 (0.0)	3 (0.6)	29 (6.1)	448 (93.3)
2.	You wear a mask when going to crowded places	0 (0.0)	0 (0.0)	35 (7.3)	445 (92.7)
3.	You wear a mask with the white side facing you and the colored side facing outwards	1 (0.2)	5 (1.0)	29 (6.1)	445 (92.7)
4.	You wear a mask by completely cover your nose, mouth, and chin	0 (0.0)	8 (1.7)	33 (6.8)	439 (91.5)
5.	You are not in close contact with the people in quarantine for COVID-19	12 (2.5)	4 (0.8)	38 (7.9)	426 (88.8)
6.	You eat clean and cooked food	1 (0.2)	5 (1.0)	53 (11.0)	421 (87.8)
7.	You avoid going to a live animal market	10 (2.1)	9 (1.9)	48 (10.0)	413 (86.0)
8.	You clean your hands: 8.1. After using the toilet	1 (0.2)	19 (4.0)	67 (14.0)	393 (81.8)
	8.2. After exposure to saliva or respiratory secretions	7 (1.5)	24 (5.0)	108 (22.5)	341 (71.0)
	8.3. After touching fresh food such as meat	10 (2.1)	32 (6,7)	100 (20.8)	338 (70.4)
	8.4. Before eating	2 (0.4)	20 (4.2)	14 (30.4)	312 (65.0)
	8.5. After coughing and sneezing	15 (3.1)	51 (10.6)	174 (36.3)	2 40 (50.0)
	8.6. After touching or hugging people	21 (4.4)	67 (14.0)	177 (36.8)	215 (44.8)
	8.7. After taking off a mask	19 (4.0)	64 (13.3)	188 (39.2)	209 (43.5)
	8.8. Before putting on a mask	25 (5.2)	81 (16.9)	190 (39.6)	184 (38.3)
9.	You avoid going to live animal markets	13 (2.7)	9 (1.9)	71 (14.8)	387 (80.6)
10.	You do not touch a living animal or animal carcasses without gloves	11 (2.3)	29 (6.0)	69 (14.4)	371 (77.3)
11.	You avoid public transportation	8 (1.6)	20 (4.2)	86 (17.9)	3 66 (76.3)
12.	You change the mask if it becomes wet or soiled	2 (0.4)	27 (5.6)	93 (19.4)	358(74.6)
13.	You do not share personal items with others, such as cutlery, dishes, and towels	11 (2.3)	30 (6.3)	102 (21.2)	337 (70.2)
14.	You throw the soiled mask in a covered trash can	7 (1.5)	34 (7.1)	104 (21.6)	335 (69.8)
15.	You throw tissue paper used to cover the mouth and nose when coughing and sneezing in a covered trash can	10 (2.1)	32 (6.7)	114 (23.7)	324 (67.5)
16.	You keep your body warm	4 (0.8)	17 (3.6)	148 (30.8)	311 (64.8)
17.	You use a serving spoon when sharing food	9 (1.9)	45 (9.4)	139 (29.0)	287 (59.7)
18.	You avoid physical contact with quarantined people	24 (5.0)	50 (10.5)	137 (28.5)	269(56.0)
19.	You avoid touching or hugging people	3 (0.6)	19 (4.0)	177 (36.9)	281 (58.5)
20.	You avoid using crowded elevators	5 (1.0)	31 (6.5)	179 (37.3)	265 (55.2)
21.	You use at least one mask per day	7 (1.4)	81 (16.9)	128 (26.7)	264 (55.0)
22.	You study or work from home	54 (11.3)	50 (10.4)	121 (25.2)	255 (53.1)
23.	You do not touch outside the mask when taking it off	11 (2.3)	67 (14.0)	148 (30.8)	254 (52.9)
24.	You sleep at least 6 h per night	10 (2.0)	55 (11.5)	162 (33.8)	253 (52.7)
25.	You use tissue paper to cover the mouth and nose when coughing and sneezing if you do not wear a mask	16 (3.4)	50 (10.4)	173 (36.0)	241 (50.2)
26.	You keep physical distancing 1–2 m between yourself and others	1 (0.2)	38 (7.9)	201 (41.9)	240 (50.0)
27.	You wash your hands by washing the palm, back of the hands, between the fingers, under your fingernails, and wrists	1 (0.2)	55 (11.4)	187 (39.0)	237 (49.4)
28.	You avoid touching your eyes, nose, and mouth	4 (0.8)	43 (9.0)	198 (41.2)	235 (49.0)
29.	You spend 20 s for hand washing with plain soap or antiseptic soap	1 (0.2)	48 (10.0)	197 (41.0)	234 (48.8)
30.	You perform a fit check when donning an N95 mask	42 (8.8)	84 (17.4)	120 (25.0)	234 (48.8)
31.	You rub your hand by rubbing the palm, back of the hands, between the fingers, under your fingernails, and wrists if your hands are invisibly soiled	1 (0.2)	67 (14.0)	179 (37.3)	233 (48.5)
32.	You avoid touching your surrounding environment	1 (0.2)	36 (7.5)	228 (47.5)	215 (44.8)
33.	You avoid going out	13 (2.6)	58 (12.1)	211 (44.0)	198 (41.3)
34.	You wear gloves before cleaning surfaces or dirt	28 (5.8)	104 (21.7)	165 (34.4)	183 (38.1)
35.	You clean frequently touched surfaces, such as beds, tables, chairs, and objects around the bathroom, with bleach	33 (6.9)	110 (22.9)	186 (38.7)	151 (31.5)

**Table 4:** Factors Predicting Practices in Prevention of COVID-19 among participants (*n* = 480).

Factors	Wearing a Mask and Coughing Etiquette			Practicing Hand Hygiene			Avoiding Crowded Places			Practicing Health Promotion			Overall Prevention Practice		
	B	Beta	*p*-Value	B	Beta	*p*-Value	B	Beta	*p*-Value	B	Beta	*p*-Value	B	Beta	*p*-Value
Constant	9.72	-	<0.001	4.89	-	0.048	3.22	-	0.004	13.01	-	<0.001	32.01	-	<0.001
Female	-	-	-	1.93	0.15	<0.001	0.56	0.11	0.011	-	-	-	3.66	0.11	0.006
Age	0.04	0.14	0.002	-	-	-	0.02	0.11	0.049	-	-	-	-	-	-
Student	-	-	-	-	-	-	0.99	0.22	0.002	-	-	-	-	-	-
University staff	-	-	-	-	-	-	−0.83	−0.18	0.001	-	-	-	-	-	-
Patient	-	-	-	1.74	0.16	<0.001	-	-	-	2.39	0.20	<0.001	4.93	0.17	<0.001
Knowledge	-	-	-				−0.17	−0.23	<0.001	−0.23	−0.12	0.008	−0.49	−0.10	0.020
Attitude	0.14	0.30	<0.001	0.14	0.25	<0.001	0.08	0.32	<0.001	0.21	0.33	<0.001	0.56	0.37	<0.001
Perception *	0.19	0.23	<0.001	0.14	0.14	0.002	-	-	-	0.22	0.20	<0.001	0.57	0.21	<0.001
Adjusted R^2^	0.218			0.157			0.219			0.256			0.288		

* Perceived information about COVID-19.

**Table 5:** Impact of COVID-19 among participants (*n* = 480).

Score	Level	*n*	%
Overall impact of COVID-19			
13–26	Low	72	15
27–39	Moderate	226	47.1
40–52	High	182	37.9
Mean = 36.0, SD = 8.9; Median = 36.0, Range = 13–52			
Economic impact			
4–7	Low	37	7.7
8–11	Moderate	123	25.6
12–16	High	320	66.7
Mean = 12.4, SD = 3.2; Median = 13.0, Range = 4–16			
Psychological impact			
4–7	Low	62	12.9
8–11	Moderate	159	33.1
12–16	High	259	54
Mean = 11.3, SD = 3.3; Median = 12.0, Range = 4–16			
Social impact			
2–3	Low	90	18.8
4–5	Moderate	159	33.1
6–8	High	231	48.1
Mean = 5.3, SD = 1.9; Median = 5.0, Range = 2–8			
Physical impact			
3–5	Low	125	26
6–8	Moderate	176	36.7
9–12	High	179	37.3
Mean = 7.0, SD = 2.7; Median = 7.0, Range = 3–12			

## Data Availability

The original contributions presented in the study are included in the article/Supplementary Material, further inquiries can be directed to the corresponding author.

## Supplementary Materials

The following supporting information can be downloaded at: https://www.mdpi.com/article/10.3390/medicina58040505/s1, Table S1: Response to knowledge items about COVID-19 among participants; Table S2: Response to attitude items toward COVID-19 prevention among participants; Table S3: Response to perception items about COVID-19 among participants; Table S4: Response to impact items about COVID-19 among participants.
