# Supplementary material for: Factors Predicting Practices in Prevention of COVID-19 and Impacts among Population in Chiang Mai, Thailand
Source: Medicina (Kaunas). 2022 Apr 1;58(4):505. doi: 10.3390/medicina58040505 (PMC9031495; doi:10.3390/medicina58040505)
Supplement: Supplementary file 1 [file medicina-58-00505-s001.zip › medicina-1613474-supplementary.pdf]

Supplement Table S1. Response to knowledge items about COVID-19 among participants (n=480)

Knowledge items		Correct answer	
		**n**	**%**
1.	Most common symptoms of COVID-19 are fever, dry cough, and tiredness	468	97.5
2.	Preventing the spread of the COVID-19 by wearing a mask when going out	466	97.1
3.	Surveillance and observing the possible illnesses of COVID-19 for 14 days	461	96.0
4.	If a mask do not fit snugly against the sides of the face and have gaps increase risk of COVID-19	431	89.8
5.	Washing hand frequently reduces risk of COVID-19	428	89.2
6.	Avoiding traveling to high risk country reduces risk of COVID-19	428	89.2
7.	Quarantine a person who is close contact to COVID-19 patient for 14 days	425	88.5
8.	Avoiding time in crowded public areas and enclosed spaces such as elevators, and public bus reduces risk of COVID-19	415	86.5
9.	Wearing an N95 mask when caring for suspected or confirmed COVID-19 patients in healthcare settings	413	86.0
10.	Physical distancing of at least 1meter lowers risk of COVID-19 transmission	406	84.6
11.	Spending more than 2 hours in an indoor space with someone who has COVID-19 is a high risk for COVID-19 infection	399	83.1
12.	Regular household cleaning and disinfection products will effectively eliminate the virus from household surfaces	378	78.8
13.	Every day either wash a mask if it's a fabric mask, or dispose of a surgical mask in a covered trash bin	374	77.9
14.	Touching the outside of a mask increases risk of covid-19	374	77.9
15.	Performing handwashing after take off a surgical or cloth mask	349	72.7
16.	Spending 20-30 seconds for washing hand with plain soap or antiseptic soap	340	70.8
17.	Performing a fit check when donning an N95 mask	280	58.3
18.	If a tissue is not available, cough or sneeze into your elbow	253	52.7
19.	If hands are visibly soiled or contaminated with saliva or respiratory secretions, cannot use an alcohol-based hand rub	205	42.7
20.	Patients under investigation for COVID-19 may spread coronavirus to other people	200	41.7

Supplement Table S2. Response to attitude items toward COVID-19 prevention among participants (n=480)

Attitude items		Level of attitude			
		Strongly disagree	Disagree	Agree	Strongly agree
		n(%)	n(%)	n(%)	n(%)
1.	You think that wearing the mask correctly reduces the risk of COVID-19	1(0.2)	3(0.6)	70(14.6)	406(84.6)
2.	You think that wearing a mask when going to crowded places reduces the risk of COVID-19	0(0.0)	5(1.0)	74(15.5)	401(83.5)
3.	You think that avoiding the elevator that is crowded reduces the risk of COVID-19	2(0.4)	4(0.8)	75(15.7)	399(83.1)
4.	You think cleaning your hands with alcohol gel properly reduces the risk of COVID-19	0(0.0)	2(0.4)	80(16.7)	398(82.9)
5.	You think that cleaning your hands with plain soap or antiseptic soap for at least 20 seconds reduces the risk of COVID-19	0(0.0)	1(0.2)	82(17.1)	397(82.7)
6.	You think that avoiding the public transportation that is crowded helps to reduces the risk of COVID-19	5(1.0)	6(1.3)	84(17.5)	385(80.2)
7.	You think eating clean and cooked food reduces your risk of COVID-19	1(0.2)	7(1.5)	93(19.3)	379(79.0)
8.	You think that sharing personal items with others, such as cutlery, dishes, towels, increases the risk of COVID-19	7(1.5)	6(1.3)	90(18.8)	377(78.4)
9.	You think that avoiding going out reduces the risk of COVID-19	2(0.4)	6(1.3)	106(22.0)	366(76.3)
10.	You think that avoiding touching other people's bodies reduces the risk of COVID-19	1(0.2)	5(1.0)	111(23.2)	363(75.6)
11.	You think that avoiding touching your eyes, nose, and mouth reduces the risk of COVID-19	5(1.0)	7(1.5)	109(22.7)	359(74.8)
12.	You think that studying or working from home reduces the risk of COVID-19	3(0.6)	9(1.9)	117(24.4)	351(73.1)
13.	You think that physical distancing 1-2 meters between yourself and others reduces your risk of COVID-19	0(0.0)	9(1.9)	121(25.2)	350(72.9)
14.	You think that being close contact with the people in quarantine for COVID-19 increases the risk of COVID-19	7(1.5)	11(2.3)	113(23.5)	349(72.7)
15.	You think that wearing gloves before cleaning surfaces or dirt reduces the risk of COVID-19	1(0.2)	7(1.5)	127(26.4)	345(71.9)
16.	You think that avoiding going to markets that sell carcasses reduces the risk of COVID-19	9(1.9)	13(2.7)	113(23.5)	345(71.9)
17.	You think that avoiding touching your surrounding environment reduces the risk of COVID-19	1(0.2)	6(1.3)	129(26.8)	344(71.7)
18.	You think that cleaning frequently touched surfaces such as beds, tables, chairs, objects around the bathroom, with bleach reduces the risk of COVID-19	1(0.2)	12(2.5)	125(26.0)	342(71.3)
19.	You think that touching a living animal or animal carcasses without gloves increased risk of COVID-19	2(0.4)	19(4.0)	123(25.6)	336(70.0)
20.	You think that avoiding going to live animal market reduces the risk of COVID-19	4(0.8)	20(4.2)	121(25.2)	335(69.8)

21.	You think that eating food promptly and use a serving spoon when sharing food reduces the risk of infection COVID-19	14(2.9)	35(7.3)	104(21.7)	237(68.1)
22.	You think that throwing tissue paper used to cover the mouth and nose when coughing and sneezing in a covered trash reduces the risk of COVID-19 spreads	10(2.1)	13(2.7)	140(29.2)	317(66.0)
23.	You think that keeping your body warm reduces the risk of COVID-19	3(0.6)	34(7.1)	187(39.0)	256(53.3)
24.	You think that getting at least 6 hours of sleep per night reduces the risk of COVID-19	5(1.0)	46(9.6)	180(37.5)	249(51.9)
25.	You think that if a tissue is not avialable, coughing or sneezeing into your elbow the risk of COVID-19 spreads	26(5.4)	61(12.7)	155(32.3)	238(49.6)
26.	You think that using tissue paper to cover your mouth and nose when coughing and sneezing reduces the risk of COVID-19 spreads	23(4.8)	62(12.9)	168(35.0)	227(47.3)

Supplement Table S3. Response to perception items about COVID-19 among participants (n=480)

Perception items		Level of perception correct information			
		Never	Rarely	Sometimes	Always
		n(%)	n(%)	n(%)	n(%)
**Information about COVID-19**					
1.	Preventative measures	0(0.0)	3(0.6)	98(20.4)	379(79.0)
2.	Mode of transmission	0(0.0)	9(1.9)	124(25.8)	347(72.3)
3.	List of high risk countries /areas	1(0.2)	16(3.3)	135(28.2)	328(68.3)
4.	Signs and symptoms	1(0.2)	22(4.6)	135(28.1)	322(67.1)
5.	Severity of disease	3(0.6)	12(2.5)	155(32.3)	310(64.6)
6.	Surveillance	0(0.0)	15(3.1)	159(33.1)	306(63.8)
7.	Screening	3(0.6)	25(5.2)	160(33.3)	292(60.9)
8.	Treatment	3(0.6)	43(9.0)	160(33.3)	274(57.1)
9.	Outbreak situation	0(0.0)	5(1.0)	208(43.4)	267(55.6)
10.	Daily new cases	1(0.2)	25(5.2)	189(39.4)	265(55.2)
11.	Daily recovery cases	2(0.4)	25(5.2)	192(40.0)	261(54.4)
12.	Number of confirmed cases	1(0.2)	13(2.7)	224(46.7)	242(50.4)
13.	Number of patient under investigation	5(1.0)	45(9.4)	214(44.6)	216(45.0)
**Source of information**					
1.	Thailand Department of Disease Control website	13(2.7)	28(5.8)	121(25.2)	318(66.3)
2.	World Health Organization website	18(3.8)	38(7.9)	122(25.4)	302(62.9)
3.	Television	2(0.4)	21(4.4)	208(43.3)	249(51.9)
4.	Healthcare personnel	19(4.0)	32(6.6)	214(44.6)	215(44.8)
5.	Newspaper	37(7.7)	93(19.4)	221(46.1)	129(26.9)
6.	Radio	24(5.0)	96(20.0)	232(48.3)	128(26.7)
7.	Facebook	35(7.3)	116(24.1)	202(42.1)	127(26.5)

8.	Youtube	31(6.5)	118(24.6)	230(47.9)	101(21.0)
9.	Line application	42(8.8)	135(28.1)	207(43.1)	96(20.0)
10.	Colleague	51(10.6)	93(19.4)	260(54.2)	76(15.8)
11.	E-mail	75(15.6)	123(25.6)	219(45.6)	63(13.2)
12.	Neighbor	80(16.7)	146(30.4)	196(40.8)	58(12.1)

Supplement Table S4. Response to impact items about COVID-19 among participants (n=480)

Impact items		Level of impact			
		Low	Moderate	High	Very high
		n(%)	n(%)	n(%)	n(%)
**Economic impact**					
1.	Cost of mask	25(5.2)	58(12.1)	125(26.0)	272(56.7)
2.	Cost of hand sanitizer	25(5.2)	57(11.9)	143(29.8)	255(53.1)
3.	Lack of income	82(17.1)	71(14.8)	120(25.0)	207(43.1)
4.	Unemployment/out of work temporarily	106(22.1)	78(16.3)	113(23.5)	183(38.1)
**Psychological impacts**					
1.	Fear of infection	25(5.2)	68(14.1)	201(41.9)	186(38.8)
2.	Fear of quarantine	53(11.0)	106(22.1)	175(36.5)	146(30.4)
3.	Anxiety	54(11.3)	134(27.9)	161(33.5)	131(27.3)
4.	Stress	82(17.1)	146(30.4)	153(31.9)	99(20.6)
**Social impact**					
1.	Unable to perform normal daily activities	73(15.3)	97(20.2)	137(28.5)	173(36.0)
2.	Discrimination	131(27.3)	113(23.5)	121(25.2)	115(24.0)
**Physical impact**					
1.	Exhaustion	104(21.6)	174(36.3)	144(30.0)	58(12.1)
2.	Insufficient rest/sleep	102(21.3)	150(31.3)	172(35.7)	56(11.7)
3.	Insomnia	123(25.6)	142(29.6)	163(34.0)	52(10.8)
